# Prevalence Mapping of *Schistosoma mansoni* Among Pre-school Age Children in Rwanda

**DOI:** 10.3389/fped.2022.906177

**Published:** 2022-06-24

**Authors:** Nadine Rujeni, Jean Bosco Bayingana, Elias Nyandwi, Amans Ntakarutimana, Joseph Kagabo, Reverien Rutayisire, Eliah Shema, Philbert Kanimba, Jean Bosco Mbonigaba, Eugene Ruberanziza

**Affiliations:** ^1^School of Health Sciences, College of Medicine and Health Sciences, University of Rwanda, Kigali, Rwanda; ^2^Single Project Implementation Unit, University of Rwanda, Kigali, Rwanda; ^3^Centre for Geographic Information Systems and Remote Sensing, College of Science and Technology, University of Rwanda, Kigali, Rwanda; ^4^Neglected Tropical Diseases and Other Parasitic Diseases Unit, Rwanda Biomedical Center, Ministry of Health, Kigali, Rwanda

**Keywords:** schistosomiasis, pre-school aged children, risk factors, *Schistosoma mansoni*, Rwanda

## Abstract

*Schistosoma mansoni* is endemic in Rwanda, and control programs have been implemented with a special focus on school-age children (SAC), ignoring pre-school age children (pre-SAC) for which the actual prevalence of the disease is not well established. This study consisted of a cross-sectional quantitative mapping of the distribution of *Schistosoma mansoni* and identification of associated risk factors among pre-SAC throughout the country. The study covered all the 17 districts of Rwanda endemic for *Schistosoma mansoni*, with a total sample of 4,675 children enrolled from 80 purposively selected villages. The parasitological assessment of children’s urine and stool samples was conducted using CCA and Kato Katz methods, respectively, for infection detection. A standard questionnaire was used to collect data on the risk factors, and geospatial assessment was performed using tablets and GPS to record geographic coordinates for plotting locations on maps using ArcGIS software. The overall prevalence of *S. mansoni* infection across the surveyed areas was 24 and 0.8% by CCA and Kato-Katz, respectively. Infection was significantly associated with bathing children in open water bodies. Furthermore, pre-SAC looked after by siblings (sisters) were two times as much likely to be infected compared to those looked after by mothers. Schistosomiasis control interventions are needed for pre-SAC to limit their exposure to open water bodies with expectations of adapted chemotherapy to be availed. Community-based deworming campaigns may be the best way to ensure good treatment coverage of pre-SAC in Rwanda.

## Introduction

Schistosomiasis is a chronic parasitic infection that is caused by trematodes of the genus *Schistosoma*, transmitted through fresh water snails. It can cause vascular damage in individuals and is associated with growth retardation and impaired metabolism and cognition (1, 2). Schistosomiasis remains a public health concern and highly prevalent in low and middle-income countries, including Rwanda, mostly affecting poor communities with no access to potable water (3). Globally, more than 250 million people are affected by schistosomiasis, and sub-Saharan Africa accounts for 90% of all these infection burdens (4, 5). Control interventions mainly rely on regular targeted treatments (using Praziquantel) of populations in endemic areas, coupled with health education. Although the disease is targeted (as per the WHO road map) for elimination as a public health problem (defined as prevalence of <1% of heavy-intensity schistosomiasis infections) by 2030 (6), control interventions do not currently reach all populations in need in most endemic areas.

For many years, pre-school-aged children (pre-SAC) have been left out in most schistosomiasis treatment programs. This was partly due to the assumption that they are not at great risk of contracting schistosomiasis. However, considerable advances have been made in this regard, and a number of studies have shown that most of the pre-SAC in endemic areas are significantly exposed to schistosomiasis in many countries (7–14). This has prompted the WHO to formulate a new guideline, including recommendations to include pre-SAC aged 2 years+ in preventive chemotherapy, while those below 2 years should be treated on an individual basis (15).

With the realization that pre-SAC are in need of treatment came a number of challenges, including the absence of an appropriate child-friendly formulation of Praziquantel and the lack of information on the real burden of schistosomiasis in this young age group in some countries. Indeed, inclusion of pre-SAC in schistosomiasis control programs should be justified by quantification of the burden of infection in that age group in each country for optimal intervention. Furthermore, an assessment of their accessibility and local acceptability should guide implementation strategies.

Rwanda is among the countries where *S. mansoni* is endemic. It has been proved by three nationwide schistosomiasis mapping and routine health data that, in Rwanda, only *S. mansoni* is prevalent and no *Schistosoma haematobium* has ever been detected. However, the burden of schistosomiasis among pre-SAC is not well documented. Our own study on Nkombo Island in the western part of the country reported an overall prevalence of 9.5% (9), but a countrywide assessment of the disease in this age group is necessary to better inform the control program.

The Rwandan Ministry of Health has recently introduced a community-based deworming strategy whereby community health workers (CHWs) and community leaders coordinate MDAs with minimal supervision from the central level. Such a deworming strategy should be suitable for treating schistosomiasis among pre-SAC. However, this should be guided by a detailed map of the disease distribution in the country.

In light of the above, this study was conducted to estimate the prevalence, identify risk factors, and produce an illustrative, countrywide map of *S. mansoni* distribution in all moderate- to high-risk areas of the country.

## Materials and Methods

### Study Design, Population, and Sample Size

This was a cross-sectional quantitative study that involved parasitological assessment of schistosomiasis among children aged 7–68 months. Structured interviews were also held with the participants’ parents/caregivers to assess risk factors of infection in this young population. The study covered all the 17 districts of Rwanda at moderate to high risk of schistosomiasis (16, 17) and targeted 40 sectors reported with prevalence of 10% and above among school children (based on incidence and mapping data) (16, 18). From each targeted sector, 2 villages were selected based on their proximity to existing water bodies and/or wetlands. Because of the focal distribution of schistosomiasis, such purposive samplings have been recommended in mapping studies to better guide interventions toward schistosomiasis elimination (19, 20). In our study, the villages layer was intersected with layers of lakes, multipurpose water dams, fish ponds, and/or important wetlands (wetland of ≥0.7 ha hosting a socioeconomic activity, mostly irrigated agriculture). From eligible villages (high-risk villages), final selection was purposively decided, considering the spatial distribution pattern analyzed visually by researchers and complemented with field verification, making 80 study villages with proximity to open water sources and wetlands. A total of 4,675 children were randomly selected and enrolled from the purposively selected villages.

### Parasitological Assessment

Urine samples were collected and tested for schistosomiasis-circulating antigen using the Point-Of-Care Circulating Cathodic Antigen (POC-CCA or CCA), following the manufacturer’s instructions. CCA test results were recorded as negative (− or trace) or positive (+; ++ or +++ according to the intensity of the test line in comparison to a test control). Stool samples were also collected and tested using the Kato Katz method for the detection of patient infection, following published protocols (21). A single specimen was collected from which 2 slides were prepared and read by 2 laboratory technicians independently. Ten per cent (10%) of all the slides were retested by the National Reference Laboratory senior technicians for quality control. The POC-CCA estimate was considered as the overall prevalence, given the high sensitivity of the assay (16). However, KK results were considered for infection intensity, where raw fecal worm egg count (FWEC) was multiplied by 24 to estimate the eggs per gram (epg) (21, 22). The level of infection intensity was determined based on WHO guidelines (23).

### Risk Factors and Spatial Assessment

A standard questionnaire was designed in the Kobo toolbox and used to collect data from parents/guardians by a well-trained data collector using a tablet. Geographic coordinates were recorded using tablets, and ArcGIS software version 10.4 was used to produce maps.

### Statistical Analysis

Data collected were analyzed by Stata 13 (STATA Corp., College Station, TX, United States). Descriptive analysis was conducted. Categorical variables were presented as frequencies and their respective percentages. Numerical variables were presented as mean and standard deviations. For comparison purposes, cross-tabulation analyses were conducted, and contingency tables were produced, while chi-square tests were used to compare proportions. To assess the association between variables, logistic regression was performed. For binary logistic regression, each risk factor was assessed with the outcome (infection status) separately, while, for multivariable logistic regression, a backward stepwise logistic regression was run, and all risk factors with a *p*-value less or equal to 0.05 were considered in the model. Odds ratios and the corresponding 95% confidence interval (CI) were reported. A two-tailed significance level of 0.05 was considered in all analyses, and only those variables that were significant in bivariate analysis were included in multivariate analysis.

## Results

### Demographic Characteristics of the Study Participants

The total sample was 4,675 pre-SAC, with a mean age of 37.5 months (±13.0), and a sex ratio M:F of 1.01: 1 (Table 1). Majority of the study participants (corresponding to 96.2%) were born in their respective villages (permanent residents).

**TABLE 1:** Characteristics of pre-school aged children enrolled in the study.

	*n*	%
Age in months (Mean, SD) (37.5, 13.0)		
**Age categories (in months)**		
12 months and less	73	1.6
13–24 months	842	18
25–36 months	1230	26.3
37–48 months	1359	29.1
49 months and above	1171	25
Total	4675	100
**Sex**		
Female	2317	49.6
Male	2358	50.4
Total	4675	100
**Child born in village**		
No	177	3.8
Yes	4498	96.2
Total	4675	100

### Water Contact Activities (Exposure) for Children in Study Areas

More than 90% parents reported farming as their occupation, and 83.3% (*n* = 3,895) of the families use open water sources (lakes, dams, or swamps) for daily domestic activities. This results in more than 60% of enrolled children being regularly in contact with open water sources (Table 2).

**TABLE 2:** Water contact activities for parents and their children in the study areas.

	*n*	%
**Parent’s occupation**		
Casual worker	143	3.18
Farmer	4,060	90.26
Government employee	52	1.16
Self employed	157	3.49
Unemployed	86	1.91
Total	4,498	100
**Fetching water in the open water source**		
No	780	16.7
Yes	3895	83.3
Total	4675	100
**Child taken to open water source**		
No	1444	30.9
Yes	3222	69.1
Total	4666	100
**Frequency child taken to lake**		
Once a day	2055	63.4
Once a week	824	25.4
Once a month	185	5.7
Once a year	53	1.6
Once in his life	123	3.8
Total	3240	100
**Child bathed in the lake**		
No	1669	36.2
Yes	2937	63.8
Total	4606	100
**Frequency child bathed IN the lake**		
Once a day	1943	65.2
Once a week	761	25.5
Once a month	155	5.2
Once a year	38	1.3
Once in his life	83	2.8
Total	2980	100
**Child ever bathed using FETCHED lake water**		
No	947	20.3
Yes	3728	79.7
Total	4675	100

### Prevalence and Infection Intensity of Schistosomiasis Among Pre-school-Aged Children

Prevalence of schistosomiasis among pre-school-aged children was assessed using both KK and POC-CCA. The POC-CCA results yielded prevalence of 24% among children tested (trace results considered negative), while the KK only yielded 0.8% prevalence. Based on WHO guidelines, 16.2% (*n* = 6) of the 37 children who tested positive on Kato Katz had moderate infection, while the rest had light infection (Table 3).

**TABLE 3:** Prevalence and infection intensity of the study population.

	*n*	%
**POC-CCA test**		
Negative	3554	76
Positive	1121	**24**
Total	4675	100
**KK test results**		
Negative	4638	99.2
Positive	37	0.8
Total	4675	100
**Intensity of infection**		
Light	31	83.8
Moderate	6	16.2
Heavy	0	0
Total	37	100

*Bold number is the percentage of infected children (prevalence).*

### Prevalence of Schistosomiasis by Age, Gender, and Location Among Pre-school Children

The results on the prevalence of schistosomiasis infection by gender revealed no statistically significant difference in infection between males and females. However, a statistically significant difference in infection was observed across age groups, the youngest having the lowest infection burden (Table 4).

**TABLE 4:** Infection prevalence per age category and gender.

		CCA – traces considered negative		KK	
Age		Negative	Positive	Negative	Positive
≤12 months	*n*	52	21	73	0
	%	1.5	**1.9**	1.6	**0.0**
13–24 months	*n*	617	225	841	1
	%	17.4	**20.1**	18.1	**2.7**
25–36 months	*n*	915	315	1222	8
	%	25.7	**28.1**	26.3	**21.6**
37–48 months	*n*	1068	291	1346	13
	%	30.1	**26.0**	29.0	**35.1**
≥49 months	*n*	902	269	1156	15
	%	25.4	**24.0**	24.9	**40.5**
		χ^2^ (*P*-value) = 11.7493 (**0.019**)		χ^2^ (*P*-value) = 9.7921 (**0.044**)	
**Gender**					
Female	*n*	1782	535	2298	19
	%	50.1	47.7	49.5	51.4
Male	*n*	1772	586	2340	18
	%	49.9	52.3	50.5	48.6
		χ^2^ (*P*-value) = 1.9890 (0.158)		χ^2^ (*P*-value) = 0.0478 (0.827)	

*Bold numbers are the percentages of infected children in each age group. Significant p-values (for X2 tests comparing age groups).*

**FIGURE 1:**
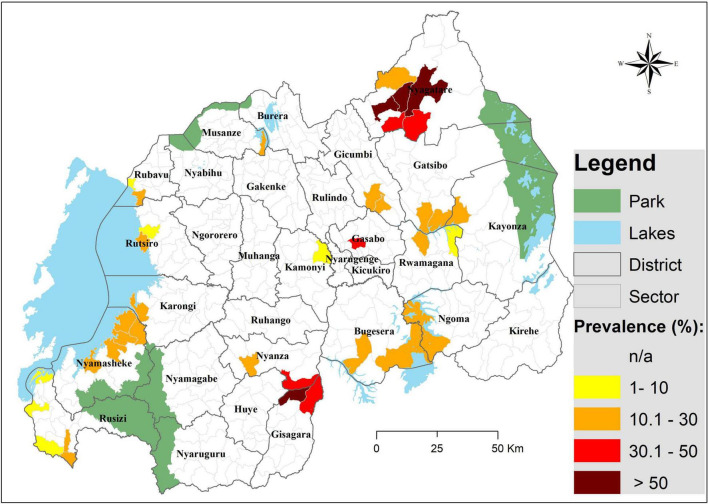
A map illustrating the distribution of *Schistosoma mansoni* infection among pre-SAC in Rwanda. Infection prevalence was based on CCA findings (trace as negative); geographical coordinates were collected for each site (village) and were extrapolated to the lowest administrative boundary (sector).

There were significant differences in infection prevalence across study sites, Gisagara district having the highest prevalence, with 60.4%, followed by Nyagatare and Gasabo districts, respectively, with 46.8 and 40%, while Kamonyi district had the lowest prevalence (7.1%). Infection distribution across study sites is illustrated on Figure 1.

### Factors Associated With Schistosomiasis Infection

The logistic regression performed highlighted the age as a contributing factor to infection status as expected. In addition, two behavioral factors were significantly associated with the occurrence of infection (Table 5): children taken care of by a sister, and those who are bathed in the lake/pond were approximately 2 times more at risk of being infected (OR = 1.9, *p* < 0.0001 and OR = 1.8, *p* < 0.0001, respectively) compared to their counterparts.

**TABLE 5:** Association between behavioral factors and schistosomiasis.

CCA_Results	Odds ratio	Std. Err.	*P*	95% Conf.	Interval
**Age group**					
≤12 months	REF				
13–24 months	0.80	0.21	0.39	0.48	1.34
25–36 months	0.72	0.18	0.20	0.43	1.19
37–48 months	0.58	0.15	0.03	0.35	0.96
≥49 months	0.60	0.16	0.05	0.36	1.01
**Child carer**					
Mum	REF				
Dad	0.81	0.13	0.19	0.58	1.11
Sister	1.91	0.32	**<0.0001**	1.37	2.66
Brother	0.37	0.20	0.07	0.13	1.07
Other	0.50	0.06	**<0.0001**	0.40	0.63
**Child bathed in Lake**					
No	REF				
Yes	1.82	0.15	**<0.0001**	1.56	2.14

*Bold numbers indicate significant p-values.*

## Discussion

The present study aimed to estimate the burden of schistosomiasis among pre-SAC in Rwanda and risk factors for the infection. Overall, the prevalence of *Schistosoma mansoni* infection was 24% using the CCA diagnostic method and 0.8% using the KK method. The high positivity of the CCA compared to KK has well been documented in other settings (24–26), and this may be related to the fact that pre-patent and single-sex infections are detected by CCA as opposed to KK, which only detects eggs in stool (26, 27). This is more relevant in pre-SAC, who are most likely carrying pre-patent and/or light infections (27). However, although there is a lack of a more sensitive and more specific field diagnostic tool for Schistosomiasis mapping, one of the limitations of the CCA is the possibility of false positives in low endemic settings (28). Nevertheless, our surveyed areas were known to have a moderate to high prevalence of *S. mansoni*, which would limit the number of false positives.

The CCA prevalence detected was comparable to findings among SAC in the same areas (16), highlighting an urgent need to include these children in treatment campaigns. As expected, the distribution of infection was skewed, few sectors harboring most of the infection burden as highlighted by the significant differences between sites (i.e., 60.5%, maximum; and 7.1%, minimum). In agreement with the previous mapping among SAC (16, 17), sectors of Nyagatare and Gisagara districts showed the highest infection prevalence (in 4 sectors, the prevalence exceeded 50%), but, also, the districts of Ngoma, Bugesera, Gatsibo, Kayonza, Nyamasheke, Gicumbi, and Karongi were heavily burdened. With regard to infection intensity, and based on WHO criteria (23), most of the pre-SAC showed light-to-moderate infection intensities.

In this study, 83.3% of the parents/guardians fetched their water for domestic use from open water sources, and 63.4% confirmed taking their children to these water sites every day. Most importantly, 63.8% of the parents/guardians interviewed bath their children in open water bodies, and our logistic regression analysis indicates that their children were two times as much likely to be infected compared to those who were not bathed in these waters. Previous studies elsewhere have also reported this passive nature of exposure to schistosomiasis among pre-SAC (10, 11, 29). Interestingly, our logistic regression highlights that children who are looked after by a sibling (a sister in this case) – most likely while their parents conduct domestic/professional chores – are two times more likely to be infected compared to those who are, constantly, with their mothers. Although we were not able to conduct a thorough observational investigation, these findings suggest that older sisters play and/or bathe their younger siblings at water contact points that are far enough from where mothers wash dishes/clothes and, therefore, far from detergents that may destroy cercaria (30) or snails (31). These detergents may – to some extent - protect those children who are, constantly, with their mothers. Another explanation could be the fact that older siblings spend more time with pre-SAC than mothers who are busy with domestic chores or income-generating activities away from water sites. It is also possible that water contact sites for older siblings are more stagnant (for playing purposes) than contact sites for mothers who need more “flowing” (and therefore “cleaner”) water for domestic use.

This study was limited in terms of depth of health education assessment as the main focus was on screening infection in the child and interviewing the caregivers to assess exposure risk factors. Nevertheless, given the findings on infection prevalence, schistosomiasis control interventions are specifically needed that focus on health education for household members’ behavior change and practices to (a) limit the children’s exposure to open water bodies and (b) improve children’s environmental sanitary conditions (32). Additional interventions, such as provision of potable water supplies for these vulnerable communities, as well as public latrines, may provide longer term solutions. Indeed, more than 90% of the interviewed parents/guardians are farmers, and most of our study sites were in close proximity to wetlands that are exploited for agriculture. Farmers generally spend several hours in the fields and, in the absence of latrines, are likely to defecate in these fields and contaminate the water bodies that are later used for bathing children. Investigations into *S. mansoni* intermediate hosts are ongoing, but preliminary results indicate that they are present in most water bodies included in our study (unpublished data).

Taken together, the present study findings stress that pre-SAC in Rwanda are part of the population at risk of schistosomiasis, and control interventions of the disease in Rwanda must include them. In particular, we provide a detailed map of pre-SAC infection distribution that should guide intervention strategies countrywide and prepare the ground for Mass Drug Administration (MDA) among pre-SAC once the Praziquantel (PZQ) pediatric formulation becomes available. Furthermore, the Rwandan Ministry of Health has recently started implementing a community-based deworming that takes place during the mother-child health (MCH) week, which should be the best way to reach pre-SAC.

Further assessment of the parents/CHW knowledge, attitudes, and practices toward schistosomiasis and WASH-recommended practices is recommended. We also recommend an integrated control of schistosomiasis in the country, including MDA, WASH interventions, and vector control.

## Data Availability Statement

The raw data supporting the conclusions of this article will be made available by the authors, without undue reservation.

## Ethics Statement

The studies involving human participants were reviewed and approved by the Institutional Review Board (IRB) of the University of Rwanda, College of Medicine and Health Sciences (Approval Notice No. 148/CMHS/IRB/2019). Written informed consent to participate in this study was provided by the participants’ legal guardian/next of kin.

## Author Contributions

NR and ER designed and coordinated the study. EN coordinated the geographical mapping of study sites and produced the map. JB, AN, JM, PK, JK, and ES conducted the field work and parasitological analyses. JB and RR conducted the statistical analyses. JB drafted the manuscript. NR secured funding and critically reviewed the manuscript. All authors had reviewed the manuscript before submission.

## Author Disclaimer

The views expressed in this publication are those of the author(s) and not necessarily those of the NIHR or the Department of Health and Social Care.

## Conflict of Interest

The authors declare that the research was conducted in the absence of any commercial or financial relationships that could be construed as a potential conflict of interest.

## Publisher’s Note

All claims expressed in this article are solely those of the authors and do not necessarily represent those of their affiliated organizations, or those of the publisher, the editors and the reviewers. Any product that may be evaluated in this article, or claim that may be made by its manufacturer, is not guaranteed or endorsed by the publisher.
